# Synergistic Antitumor Effects of Rosmarinic Acid and Cisplatin in Retinoblastoma: Evidence from 2D and 3D Tumor Models

**DOI:** 10.3390/biomedicines14030602

**Published:** 2026-03-08

**Authors:** Erkan Duman, Aydın Maçin, İlhan Özdemir, Şamil Öztürk, Mehmet Cudi Tuncer

**Affiliations:** 1 Department of Ophthalmology, WestEye Private Hospital, Erbil 44001, Iraq; ophthalmo48@outlook.com; 2 Department of Ophthalmology, Diyarbakır Private Batı Hospital, 21100 Diyarbakır, Turkey; draydinmacin@outlook.com; 3 Department of Histology and Embryology, Faculty of Medicine, Kahramanmaraş Sütçü İmam University, 46040 Kahramanmaraş, Turkey; ilhanozdemir25@yandex.com; 4 Vocational School of Health Services, Çanakkale Onsekiz Mart University, 17100 Çanakkale, Turkey; ozturksamil@outlook.com; 5 Department of Anatomy, Faculty of Medicine, Dicle University, 21280 Diyarbakır, Turkey

**Keywords:** retinoblastoma, Y79, WERI-Rb1, rosmarinic acid, cisplatin, synergy, apoptosis, chemotherapy

## Abstract

**Background/Objectives**: Retinoblastoma (RB) is the most common primary intraocular malignancy in children, with treatment limited by chemoresistance and therapy-related toxicity. Enhancing the efficacy of conventional chemotherapeutics while reducing dose-related adverse effects is crucial. This study investigates the chemosensitizing potential of rosmarinic acid (RA), a natural polyphenolic compound, in combination with cisplatin (Cis) in RB models. **Methods**: The antiproliferative and synergistic effects of RA and Cis were evaluated in Y79 and WERI-Rb1 RB cell lines using MTT assays and Combination Index (CI) analysis. Apoptosis and oxidative stress were assessed by Annexin V-FITC/PI flow cytometry and intracellular reactive oxygen species (ROS) measurements, respectively. Three-dimensional (3D) tumor spheroids were generated from Y79 cells for in vitro validation using spheroid size analysis, ATP-based viability assays, and live/dead fluorescence staining. The ROS dependency of cytotoxicity was further examined using N-acetylcysteine (NAC) pretreatment. Cytokine secretion was analyzed by ELISA, and apoptosis-related gene expression was assessed by qRT-PCR. **Results**: RA and Cis reduced cell viability in a dose- and time-dependent manner, while their combination induced significantly enhanced cytotoxicity, confirmed by CI values < 1. Combined treatment increased apoptotic populations, elevated intracellular ROS, and upregulated Caspase-3 and Caspase-9. These effects were maintained in 3D spheroids, with reduced spheroid size and impaired integrity. NAC pretreatment attenuated ROS generation and partially rescued cell viability, indicating a ROS-dependent, but not exclusive, contribution to cytotoxicity. **Conclusions**: RA synergistically enhances cisplatin-induced anticancer effects in RB through oxidative stress, engagement of intrinsic (mitochondria-associated) apoptotic signaling, and reduction of tumor cell-derived inflammatory and angiogenic mediators. These findings highlight the potential of RA and Cis combination as a chemosensitizing strategy for RB therapy, warranting further in vivo evaluation.

## 1. Introduction

RB is the most common primary intraocular malignancy in children and accounts for the majority of intraocular tumors diagnosed during early childhood [1,2]. Although significant advancements have been achieved in early diagnosis and multimodal management including systemic chemotherapy, focal therapies, radiotherapy, and enucleation, substantial challenges remain. In advanced disease stages, chemoresistance, treatment-associated toxicity, and the risk of metastatic spread continue to limit therapeutic success and adversely affect long-term survival outcomes [3,4].

Current systemic chemotherapy protocols for retinoblastoma commonly rely on multi-agent regimens, particularly carboplatin-based combinations, frequently administered intravenously or via intra-arterial chemotherapy [4,5]. Although cisplatin is not considered a standard first-line agent in contemporary RB treatment protocols, platinum-based compounds constitute a mechanistically relevant class of DNA-damaging chemotherapeutics widely used in solid tumors.

In the present study, Cis was selected as a prototypical platinum-based compound with a well-characterized mechanism of DNA damage induction and a reproducible in vitro cytotoxic profile. Its use in this experimental context does not imply clinical preference over currently established carboplatin-based regimens in retinoblastoma, but rather provides a mechanistically relevant model to investigate platinum-based chemosensitization strategies. Furthermore, future investigations should extend these findings to clinically utilized agents such as carboplatin to enhance translational relevance and better reflect current therapeutic standards.

Despite the clinical utility of platinum-based agents, their therapeutic efficacy may be limited by the development of multidrug resistance and dose-dependent systemic toxicity [5]. These limitations underscore the need for adjunct strategies capable of enhancing anticancer efficacy while reducing adverse effects. In this context, combination approaches aimed at modulating oxidative stress and apoptotic signaling pathways represent a rational strategy to improve platinum-based therapy.

RA is a naturally occurring polyphenolic compound widely distributed in plants of the Lamiaceae family, including rosemary, sage, thyme, and basil. RA exhibits diverse biological activities, including antioxidant, anti-inflammatory, anti-angiogenic, and anticancer effects [6,7]. Previous studies have demonstrated that RA can inhibit tumor cell proliferation, induce apoptosis, regulate intracellular ROS levels, and suppress inflammatory and angiogenic mediators across a range of cancer types, such as breast, colon, lung, hepatic, and prostate cancers [8]. Importantly, RA has also been reported to synergize with conventional chemotherapeutic agents, thereby enhancing anticancer efficacy and potentially overcoming drug resistance mechanisms [6,8].

Cisplatin exerts its antitumor activity primarily through the formation of DNA adducts and intra- and interstrand crosslinks, thereby interfering with DNA replication and repair and ultimately triggering apoptotic cell death. In addition to DNA damage, cisplatin is associated with oxidative stress generation and activation of intrinsic apoptotic pathways [9].

Despite these promising properties, the therapeutic potential of RA in RB, particularly in combination with cisplatin, remains largely unexplored. Given the aggressive nature of RB and the multifactorial mechanisms underlying chemoresistance, oxidative stress imbalance, and tumor cell-derived inflammatory signaling, effective combination strategies targeting these interconnected pathways are urgently needed.

Intravenous chemotherapy is available in the vast majority of retinoblastoma centers worldwide, and the most commonly employed frontline regimen consists of carboplatin, etoposide, and vincristine (CEV), which has demonstrated high ocular salvage rates in early- and intermediate-stage disease and remains the backbone of contemporary systemic RB management [10,11]. Although cisplatin is not considered a standard first-line agent in retinoblastoma treatment protocols, platinum-based compounds represent a mechanistically well-characterized class of DNA-damaging agents widely used in oncology. In this context, cisplatin was selected as a prototypical platinum compound to model platinum-based chemosensitization strategies under controlled experimental conditions. Furthermore, growing evidence suggests that rosmarinic acid may attenuate chemotherapy-associated resistance and mitigate treatment-related toxicities, supporting its investigation as a complementary agent in platinum-based therapeutic settings [12].

The aim of the present study was to evaluate the individual and combined effects of Cis and RA in human Y79 and WERI-Rb1 retinoblastoma cells and to investigate the mechanistic basis of their interaction. Specifically, we examined their impact on cell viability, synergistic drug interactions, apoptosis induction, intracellular ROS generation, tumor cell-derived cytokine release, and the expression of key apoptosis-related genes in two-dimensional culture conditions. To further validate the biological relevance of the observed synergistic effects, a three-dimensional tumor spheroid model was employed to assess treatment-induced changes in spheroid morphology, size, viability, and cell death. In addition, the contribution of oxidative stress to RA- and Cis-induced cytotoxicity was functionally examined using NAC pretreatment as a ROS scavenging approach. Bioinformatics analyses and molecular docking studies were performed to provide complementary mechanistic and structural insight into the molecular pathways potentially underlying the synergistic interaction between RA and Cis. The synergistic effects of the drug combination were quantified using the Chou–Talalay method, a well-established model for the evaluation of combinational drug effects [13]. Collectively, these investigations aim to contribute to the development of more effective and potentially safer combination strategies for retinoblastoma therapy.

## 2. Materials and Methods

### 2.1. Chemicals and Reagents Used for Cell Culture, Drug Treatment, and Cellular Analyses

Rosmarinic acid (RA; ≥98% purity; Sigma-Aldrich, St. Louis, MO, USA) was dissolved in dimethyl sulfoxide (DMSO; Sigma Aldrich, St. Louis, MO, USA) to prepare a 100 mM stock solution. Stock solutions were aliquoted and stored at −20 °C until use. Cis (Cis; ≥99.9% purity; Sigma Aldrich, St. Louis, MO, USA) was used as the chemotherapeutic agent and prepared as a 1 mM stock solution in phosphate-buffered saline (PBS; Gibco, Thermo Fisher Scientific, Waltham, MA, USA). Cis stock solutions were stored at −20 °C and protected from light.

Cell culture reagents, including RPMI-1640 medium, fetal bovine serum (FBS), penicillin–streptomycin solution, and L-glutamine, were obtained from Gibco (Thermo Fisher Scientific, Waltham, MA, USA). The MTT reagent (3-(4,5-dimethylthiazol-2-yl)-2,5-diphenyltetrazolium bromide) was purchased from Sigma-Aldrich (St. Louis, MO, USA). Apoptosis analyses were performed using an Annexin V-FITC/Propidium Iodide (PI) apoptosis detection kit obtained from BD Biosciences (San Jose, CA, USA).

### 2.2. Cell Culture Conditions and Maintenance of Human Y79 RB Cells

The human RB cell line Y79 (ATCC^®^ HTB-18™; American Type Culture Collection, Manassas, VA, USA) and WERI-Rb1 (ATCC^®^ HTB-169™) were used in this study. Y79 and WERI-Rb1 cells were maintained as suspension cultures in RPMI-1640 medium supplemented with 10% FBS and 1% penicillin-streptomycin (Gibco, Thermo Fisher Scientific, Waltham, MA, USA). Cell cultures were incubated at 37 °C in a humidified atmosphere containing 5% CO_2_.

Cells at passage 5 were used for all experiments to ensure phenotypic consistency. Prior to treatment, cells were harvested by centrifugation at 1000 rpm for 5 min, the supernatant was discarded, and the cell pellet was gently resuspended in fresh complete culture medium before seeding for subsequent assays.

### 2.3. Determination of Half-Maximal Inhibitory Concentration (IC_50_) Values and Definition of Experimental Treatment Groups for Combination Studies

To determine appropriate concentrations for combination experiments, the 48 h IC_50_ values of RA and Cis were first determined individually. Y79 and WERI-Rb1 cells were seeded into 96-well plates and treated with a wide range of concentrations of RA (10–400 µM) or Cis (1–100 µM) for 24, 48, and 72 h. Cell viability was assessed using the MTT assay, and IC_50_ values were calculated graphically based on dose–response curves. For all subsequent combination experiments, IC_50_ values corresponding to the 48 h treatment period were used.

Based on these IC_50_ determinations, the following experimental groups were established and treated for 48 h:

1. Control group: Cells treated with DMSO at the maximum final concentration used in the experiments (0.1% *v*/*v*), which was confirmed to have no significant effect on cell viability.
2. Rosmarinic acid (RA) group: Cells treated with RA alone at a concentration corresponding to its 48 h IC_50_ value.
3. Cisplatin (Cis) group: Cells treated with Cis-alone at a concentration corresponding to its 48 h IC_50_ value.
4. Combination (RA + Cis) group: Cells treated simultaneously with RA and Cis, corresponding to the respective 48 h IC_50_ concentrations of each agent.

### 2.4. Determination of Cell Viability Using the MTT Colorimetric Assay Following Single and Combination Treatments

Y79 and WERI-Rb1 cells were seeded into 96-well plates at a density of 5 × 10^3^ cells per well and allowed to equilibrate prior to treatment. Cells were then exposed to the indicated single-agent or combination treatment groups for 48 h. Following treatment, MTT solution was added to each well at a final concentration of 0.5 mg/mL, and the plates were incubated at 37 °C for 4 h to allow the formation of insoluble formazan crystals.

After incubation, the culture medium was carefully removed, and DMSO was added to each well to solubilize the formazan crystals. Absorbance was measured using a microplate reader (BioTek, Winooski, VT, USA) at 570 nm, with a reference wavelength of 630 nm to correct for background interference. Cell viability was expressed as a percentage relative to the control group.

### 2.5. Quantitative Evaluation of Drug Interactions Using the Chou–Talalay CI Method

The Chou–Talalay method was applied to quantitatively evaluate the nature of the interaction between Cis and RA, including synergistic, additive, or antagonistic effects [10]. CI values were calculated using CompuSyn software (ComboSyn Inc., Paramus, NJ, USA), which is specifically designed for dose–effect and drug interaction analyses.

For CI determination, additional dose–response experiments were performed in which RA and Cis were combined at defined fractions of their respective 48 h IC_50_ values. To generate the Fa–CI relationship, the two agents were applied at a constant ratio based on their individual 48 h IC_50_ values in each cell line. Serial dilutions of this fixed-ratio combination were used to produce graded levels of growth inhibition, allowing calculation of CI values across multiple fractional effect (Fa) levels according to the median-effect principle of Chou and Talalay. Dose–effect parameters for each single agent and for the combination were entered into CompuSyn software to calculate both CI and dose reduction index (DRI) values. According to the Chou–Talalay criteria, CI < 1 indicates synergism, CI = 1 indicates additivity, and CI > 1 indicates antagonism.

### 2.6. Bliss Independence Analysis

To further evaluate the interaction between RA and Cis, Bliss independence analysis was performed using cell viability data obtained from the MTT assay. The Bliss model assumes that two agents act independently through distinct mechanisms, and the expected combined inhibitory effect (E_Bliss) was calculated as:

E_Bliss = E_A + E_B − (E_A × E_B),

where E_A and E_B represent the fractional inhibitory effects of RA and Cis alone at the corresponding concentrations.

Fractional inhibition values (E) were derived from normalized viability data using the Formula:

E = 1 − (viability of treated cells/viability of control cells).

The Bliss excess score (ΔBliss) was calculated as the difference between the experimentally observed combined effect (E_observed) and the predicted Bliss effect (E_Bliss):

ΔBliss = E_observed − E_Bliss.

A positive ΔBliss value indicates synergism, a value close to zero indicates an additive effect, and a negative value indicates antagonism. Bliss excess scores were calculated across the full concentration matrix of RA (10–400 µM) and Cis (1–100 µM), and results were visualized as heatmaps to illustrate the interaction profile across dose combinations.

### 2.7. Detection and Quantification of Apoptosis by Annexin V-FITC and PI Flow Cytometry Analysis

Y79 and WERI-Rb1 cells were seeded into 6-well plates at a density of 2 × 10^5^ cells per well and treated with the indicated single-agent or combination regimens at concentrations corresponding to their respective 48 h IC_50_ values. Following 48 h of treatment, cells were collected and processed for apoptosis analysis according to the manufacturer’s instructions provided with the Annexin V-FITC/PI apoptosis detection kit (BD Biosciences, San Jose, CA, USA).

Briefly, cells were harvested, washed, and resuspended in Annexin V binding buffer, followed by staining with Annexin V-FITC and PI. The samples were incubated for 15 min at room temperature in the dark and subsequently analyzed using a flow cytometer (BD FACSCelesta™, BD Biosciences, San Jose, CA, USA). Cell populations were classified as viable (Annexin V^−^/PI^−^), early apoptotic (Annexin V^+^/PI^−^), or late apoptotic/necrotic (Annexin V^+^/PI^+^).

Flow cytometry data were analyzed using FlowJo software (version 10.8.1; FlowJo LLC, Ashland, OR, USA).

### 2.8. Quantification of Tumor Cell–Derived Cytokine Release by ELISA

The concentrations of cytokines released into the cell culture supernatants, including interleukin-6 (IL-6), interleukin-8 (IL-8), tumor necrosis factor-alpha (TNF-α), transforming growth factor-beta (TGF-β), and vascular endothelial growth factor (VEGF), were quantified using commercially available human-specific ELISA kits (Elabscience Biotechnology Inc., Houston, TX, USA).

Following 48 h of treatment under the indicated experimental conditions, cell culture supernatants were carefully collected and centrifuged at 1000× *g* for 10 min to remove cellular debris. The clarified supernatants were then subjected to ELISA analysis performed strictly in accordance with the manufacturer’s instructions for each cytokine.

Absorbance was measured at 450 nm using a microplate reader (BioTek, Winooski, VT, USA). Cytokine concentrations were calculated from standard curves generated for each analyte and are expressed as pg/mL.

### 2.9. Measurement of Intracellular ROS Generation Using 2′,7′-Dichlorofluorescin Diacetate (DCFH-DA) Fluorescence Assay

Following the indicated treatments, intracellular ROS levels were assessed using the cell-permeable fluorescent probe DCFH-DA (DCFH-DA; Sigma-Aldrich, St. Louis, MO, USA). Briefly, treated Y79 cells were incubated with DCFH-DA at a final concentration of 10 µM for 30 min at room temperature in the dark.

After incubation, fluorescence intensity was measured using a microplate reader (BioTek, Winooski, VT, USA) with excitation and emission wavelengths set at 485 nm and 530 nm, respectively. The fluorescence intensity of oxidized dichlorofluorescein was used as an indicator of intracellular ROS levels, with increased fluorescence reflecting elevated ROS production.

### 2.10. Caspase-3/7 Activity Assay

Caspase-3/7 activity was measured to evaluate apoptosis induction in Y79 and WERI-Rb1 RB cells following treatment with RA, Cis, or their combination. Cells were seeded into white 96-well plates at a density of 1 × 10^4^ cells per well and allowed to equilibrate overnight. The following day, cells were treated with RA, Cis, or RA + Cis at their respective IC_50_ concentrations for 48 h. Untreated cells served as the control group. After treatment, caspase-3/7 activity was determined using a commercial luminescent Caspase-Glo^®^ 3/7 Assay kit (Promega, Madison, WI, USA), according to the manufacturer’s instructions. Briefly, an equal volume of Caspase-Glo^®^ 3/7 reagent was added directly to each well, and the plates were gently mixed for 30 s to ensure cell lysis. The plates were then incubated at room temperature for 30 min in the dark to allow caspase-dependent cleavage of the luminogenic substrate. Caspase-3/7 activity was expressed as relative luminescence units (RLU) and normalized to the control group. Each experimental condition was performed in triplicate, and experiments were independently repeated at least three times.

### 2.11. qRT-PCR Gene Expression Analysis

#### 2.11.1. Isolation of Total RNA and Synthesis of Complementary DNA for Gene Expression Analysis

Total RNA was isolated from Y79 RB cells following 48 h of treatment using TRIzol™ reagent (Invitrogen™, Thermo Fisher Scientific, Waltham, MA, USA) in accordance with the manufacturer’s instructions. The concentration and purity of the isolated RNA were assessed using a NanoDrop One spectrophotometer (Thermo Fisher Scientific, Waltham, MA, USA). Only RNA samples with an A260/A280 ratio between 1.8 and 2.0 were used for subsequent analyses.

For complementary DNA (cDNA) synthesis, 1 µg of total RNA from each sample was reverse-transcribed using the High-Capacity cDNA Reverse Transcription Kit (Applied Biosystems™, Thermo Fisher Scientific, Waltham, MA, USA), following the manufacturer’s recommended protocol.

#### 2.11.2. qRT-PCR Analysis of Apoptosis-Related Gene Expression

qRT-PCR analysis was performed using a SYBR Green-based detection system to evaluate the mRNA expression levels of apoptosis-related genes. Amplification reactions were carried out on a StepOnePlus™ Real-Time PCR System (Applied Biosystems™, Thermo Fisher Scientific, Waltham, MA, USA) using Power SYBR™ Green PCR Master Mix (Applied Biosystems™, Thermo Fisher Scientific, Waltham, MA, USA) and gene-specific primer sets (Table 1).

The thermal cycling conditions consisted of an initial denaturation step at 95 °C for 10 min, followed by 40 cycles of denaturation at 95 °C for 15 s and annealing/extension at 60 °C for 1 min. Relative gene expression levels were calculated using the 2^−ΔΔCt^ method. β-Actin (ACTB) was used as the internal reference gene for normalization.

The Bax/Bcl-2 mRNA expression ratio was determined by dividing the relative expression values of Bax and Bcl-2 obtained from the 2^−ΔΔCt^ calculations for each sample. The ratio in the control group was set to 1.0, and all experimental groups were normalized accordingly.

### 2.12. Bioinformatics-Based Protein–Protein Interaction Network Construction

#### 2.12.1. Kyoto Encyclopedia of Genes and Genomes (KEGG) Pathway Enrichment Analysis of Apoptosis- and Inflammation-Related Targets

Bioinformatics analyses were performed to explore potential molecular interactions and signaling pathways associated with RA and Cis treatment. The Search Tool for the Retrieval of Interacting Genes/Proteins (STRING) database (version 12.0; https://string-db.org) was used to construct a protein–protein interaction (PPI) network encompassing key apoptosis- and inflammation-related proteins. The target proteins included Bax, Bcl-2, Caspase-3, IL-6, IL-8, TNF-α, TGF-β, and VEGF.

The PPI network was generated using a high-confidence interaction score threshold of ≥0.7 to ensure reliability of predicted protein interactions. Functional pathway enrichment analysis of the selected target proteins was subsequently conducted using KEGG database. KEGG pathway analyses were performed using both the STRING platform and the Database for Annotation, Visualization, and Integrated Discovery (DAVID) bioinformatics resource to identify significantly enriched signaling pathways related to apoptosis, inflammation, and cancer-associated processes [14,15].

#### 2.12.2. Molecular Docking Analysis to Explore Potential Interactions of RA with Apoptosis- and Inflammation-Related Target Proteins

Molecular docking analyses were conducted to explore potential interactions between RA and selected proteins involved in apoptotic and inflammatory signaling pathways, including Bcl-2, caspase-3, and TNF-α. The three-dimensional crystal structures of the target proteins were retrieved from the RCSB Protein Data Bank (PDB; https://www.rcsb.org), using the following PDB identifiers: Bcl-2 (PDB ID: 1G5M), caspase-3 (PDB ID: 1GFW), and TNF-α (PDB ID: 2AZ5).

The three-dimensional structure of RA was obtained from the PubChem database (https://pubchem.ncbi.nlm.nih.gov). Molecular docking simulations were performed using AutoDock Vina software (version 1.2.3) [16]. The predicted binding conformations and interaction profiles were analyzed, and hydrogen bond interactions and binding pocket orientations were visualized using PyMOL (version 2.5.5; Schrödinger, LLC, New York, NY, USA) and Discovery Studio Visualizer (version 2021; Dassault Systèmes BIOVIA, San Diego, CA, USA).

### 2.13. Three-Dimensional Tumor Spheroid Culture and Imaging

#### 2.13.1. Formation of 3D Tumor Spheroids

Three-dimensional (3D) tumor spheroids were generated from human retinoblastoma Y79 cells using ultra-low attachment (ULA) 96-well round-bottom plates (Corning Inc., Corning, NY, USA) to better recapitulate in vivo tumor architecture and cell–cell interactions. Y79 cells were harvested during the logarithmic growth phase and resuspended in complete RPMI-1640 medium supplemented with 10% fetal bovine serum and 1% penicillin–streptomycin.

Cells were seeded at a density of 3 × 10^3^ cells per well in a final volume of 200 µL and incubated at 37 °C in a humidified atmosphere containing 5% CO_2_. Under these conditions, compact and uniformly shaped spheroids formed within 48–72 h, as confirmed by inverted light microscopy.

#### 2.13.2. Drug Treatment of 3D Spheroids

After spheroid formation, the culture medium was gently replaced with fresh medium containing RA, Cis, or their combination (RA + Cis). Drug concentrations were selected based on IC_50_ values obtained from 2D monolayer experiments and were increased by approximately 20–30% to account for reduced drug penetration in 3D culture systems.

The following experimental groups were established:

- Control (vehicle-treated)
- RA-treated
- Cis-treated
- RA + Cis-treated.

Spheroids were incubated with the respective treatments for 72 h prior to downstream analyses. Vehicle concentrations were kept constant across all experimental groups, and treatment-containing medium was not replaced during the incubation period.

#### 2.13.3. Bright-Field Imaging and Spheroid Size Quantification

Bright-field images of 3D Y79 spheroids were acquired using an inverted microscope equipped with a digital camera (Olympus IX73, Olympus Corporation, Tokyo, Japan) at 10× magnification. Images were captured using identical optical settings for all experimental groups to ensure comparability.

Spheroid size was quantified by measuring spheroid diameter using ImageJ software (version 1.54; National Institutes of Health, Bethesda, MD, USA). For each experimental condition, at least five spheroids per well from three independent experiments were analyzed, and mean spheroid diameter values were calculated.

#### 2.13.4. Cell Viability Assessment in 3D Spheroids

Cell viability within 3D Y79 tumor spheroids was assessed using an ATP-based CellTiter-Glo^®^ 3D Cell Viability Assay (Promega, Madison, WI, USA), according to the manufacturer’s instructions. Briefly, an equal volume of CellTiter-Glo^®^ 3D reagent was added directly to each well containing spheroids and incubated for 30 min at room temperature with gentle shaking to ensure complete spheroid lysis.

Luminescence was measured using a microplate reader (BioTek, Winooski, VT, USA), and cell viability was expressed as a percentage relative to control spheroids.

#### 2.13.5. Live/Dead Fluorescence Staining and Image Acquisition

Live/dead staining of 3D Y79 tumor spheroids was performed using Calcein-AM and Ethidium homodimer-1 (EthD-1) to visualize viable and non-viable cells, respectively. After treatment, spheroids were incubated with Calcein-AM (2 µM) and EthD-1 (4 µM) in phosphate-buffered saline for 30 min at room temperature in the dark.

Fluorescence images were acquired using an inverted fluorescence microscope equipped with FITC and TRITC filter sets. Images were captured at 10× magnification using a scientific CMOS camera (DP74, Olympus Corporation, Tokyo, Japan) under identical exposure conditions for all experimental groups (green channel: 200–300 ms; red channel: 300–400 ms). Images were obtained at the spheroid mid-plane using a single focal plane to ensure consistency across samples.

No post-acquisition brightness or contrast adjustments were applied. Representative images were exported at 300 dpi resolution for figure preparation.

### 2.14. Evaluation of ROS-Dependent Cytotoxicity Using NAC Pretreatment

To investigate the role of ROS in RA- and Cis-induced cytotoxicity, a rescue experiment was performed using the ROS scavenger NAC. Retinoblastoma Y79 cells were seeded under standard culture conditions and pretreated with NAC at a final concentration of 5 mM for 1 h at 37 °C prior to drug exposure.

Following NAC pretreatment, cells were treated with RA, Cis, or their combination (RA + Cis) at the same concentrations used in previous experiments. Control groups included untreated cells and cells treated with NAC alone. Cells were incubated for the indicated time periods before downstream analyses.

#### 2.14.1. Intracellular ROS Measurement

Intracellular ROS levels were quantified using DCFH-DA. After treatments, cells were incubated with DCFH-DA (10 µM) for 30 min at 37 °C in the dark, washed with phosphate-buffered saline, and analyzed by flow cytometry. Mean fluorescence intensity (MFI) values were used to quantify intracellular ROS levels. Flow cytometry was used to allow single-cell-level quantification of intracellular ROS following NAC pretreatment.

#### 2.14.2. Cell Viability Analysis Following NAC Pretreatment

Cell viability following NAC pretreatment was assessed using the MTT assay as described above. Viability values were expressed as a percentage relative to untreated control cells.

### 2.15. Statistical Analysis

All experiments were performed with at least three independent biological replicates (*n* = 3). Data are presented as the mean ± standard deviation (SD). Statistical analyses were conducted using GraphPad Prism software version 9.0 (GraphPad Software, San Diego, CA, USA).

Comparisons among multiple experimental groups were performed using one-way analysis of variance (ANOVA), followed by Tukey’s post hoc test for multiple comparisons. A *p* value of <0.05 was considered to indicate statistical significance.

## 3. Results

### 3.1. Cytotoxic Effects of RA and Cis on Y79 and WERI-Rb1 Cells Assessed by MTT Assay

**Figure 1:**
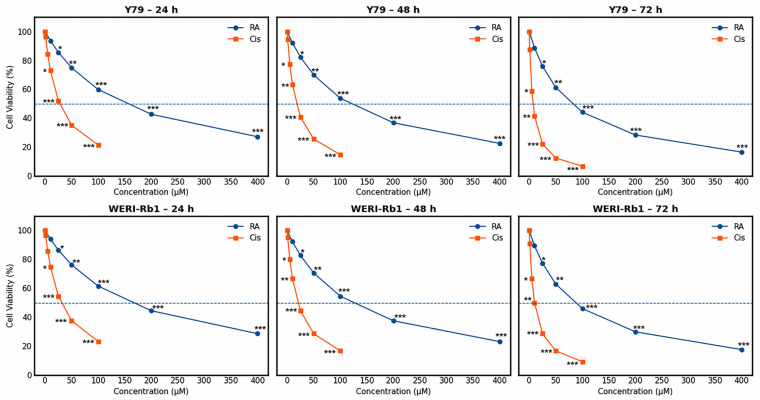
Dose–response curves of RA and Cis in Y79 and WERI-Rb1 cells at 24, 48, and 72 h. Cell viability was assessed by MTT assay and expressed as percentage of control. Each point represents the mean ± SD of three independent experiments performed in triplicate. Data were fitted using nonlinear sigmoidal regression to determine IC_50_ values. The dashed horizontal line indicates 50% viability used for IC_50_ estimation. RA exhibited moderate cytotoxicity (Y79: IC_50_ ≈ 149.1, 116.6, and 79.1 µM at 24, 48, and 72 h; WERI-Rb1: IC_50_ ≈ 159.2, 118.4, and 86.5 µM at 24, 48, and 72 h), whereas Cis demonstrated higher potency (Y79: IC_50_ ≈ 27.1, 17.2, and 7.1 µM; WERI-Rb1: IC_50_ ≈ 32.4, 18.8, and 9.5 µM at corresponding time points). Statistical significance was determined by one-way ANOVA followed by Tukey’s post hoc test (* *p* < 0.05, ** *p* < 0.01, *** *p* < 0.001 vs. control).

The cytotoxic effects of RA and Cis on Y79 and WERI-Rb1 retinoblastoma (RB) cells were evaluated using the MTT assay after 24, 48, and 72 h of treatment. Both agents induced a clear dose- and time-dependent reduction in cell viability in both cell lines (Figure 1).

In Y79 cells, RA treatment at lower concentrations (≤50 µM) produced only modest viability reductions at 24 h, with cell survival remaining above approximately 75%. However, prolonged exposure significantly enhanced its cytotoxic effect. At 72 h, higher RA concentrations (≥100 µM) reduced cell viability to approximately 15–25%, demonstrating a pronounced time-dependent increase in sensitivity. Consistently, IC_50_ values for RA progressively declined over time. Cis exhibited a markedly stronger cytotoxic effect than RA across all examined time points. Even at relatively low concentrations, Cis significantly reduced cell viability. For example, 25 µM Cis decreased viability to approximately 45% at 48 h and to below 30% at 72 h, consistent with the pronounced time-dependent reduction in IC_50_ values.

A comparable response pattern was observed in WERI-Rb1 cells. RA induced moderate growth inhibition at earlier time points, while longer exposure and higher concentrations resulted in substantial cytotoxicity. Cis again demonstrated greater intrinsic potency, with significant viability reductions at low micromolar concentrations. The progressive decrease in IC_50_ values over time in WERI-Rb1 cells further supports the exposure-dependent nature of both agents. Importantly, combined treatment with RA and Cis resulted in significantly greater growth inhibition than either agent alone in both cell lines at all examined time points (*p* < 0.001). Dose–response curves for the combination displayed a clear leftward shift relative to single-agent treatments, indicating enhanced potency. These findings are consistent with a sensitizing effect of RA on Cis-induced cytotoxicity.

Overall, the MTT results demonstrate that both RA and Cis exert dose- and time-dependent antiproliferative effects in RB cells, with Cis exhibiting greater intrinsic cytotoxicity. The enhanced growth inhibition observed under combination treatment is consistent with the interaction patterns identified by CI and Bliss independence analyses.

### 3.2. Cytotoxic Response to RA + Cis Combination Treatment

**Figure 2:**
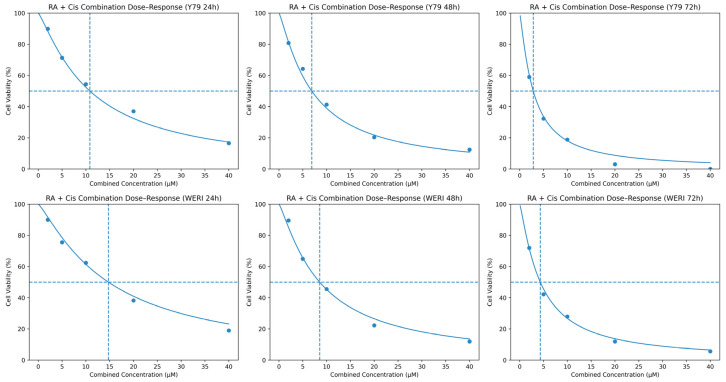
Dose–response curves of RA + Cis combination treatment in Y79 and WERI-Rb1 cells at 24, 48, and 72 h. Cell viability was assessed by MTT assay and expressed as percentage of untreated control. Data points represent mean values derived from three independent experiments performed in triplicate. Curves were fitted using nonlinear sigmoidal regression to estimate combination IC_50_ values. The dashed horizontal line indicates 50% cell viability used for IC_50_ determination. Combination treatment resulted in a time-dependent leftward shift of the dose–response curves in both cell lines, with a more pronounced effect observed at 48 and 72 h, indicating enhanced cytotoxic potency compared with single-agent treatments.

To further evaluate the cytotoxic effects of the combined RA and Cis treatment, dose–response analyses were performed using fixed-ratio combinations across increasing concentrations. As shown in Figure 2, co-treatment with RA and Cis resulted in a clear leftward shift of the dose–response curves compared with single-agent treatments in both Y79 and WERI-Rb1 cells. This shift was observed consistently at 24, 48, and 72 h, indicating enhanced potency of the combination over time. The calculated combination IC_50_ values were substantially lower than those of Cis alone at corresponding time points, particularly at 48 and 72 h, demonstrating a pronounced time-dependent increase in sensitivity. Notably, Y79 cells exhibited a greater leftward shift compared with WERI-Rb1 cells, consistent with their higher responsiveness to combination treatment. These findings indicate that RA enhances the cytotoxic efficacy of Cis in RB cells and provides the basis for subsequent quantitative interaction analyses using CI and Bliss independence models.

### 3.3. Evaluation of Drug Interaction Between RA and Cis Using CI Analysis

**Figure 3:**
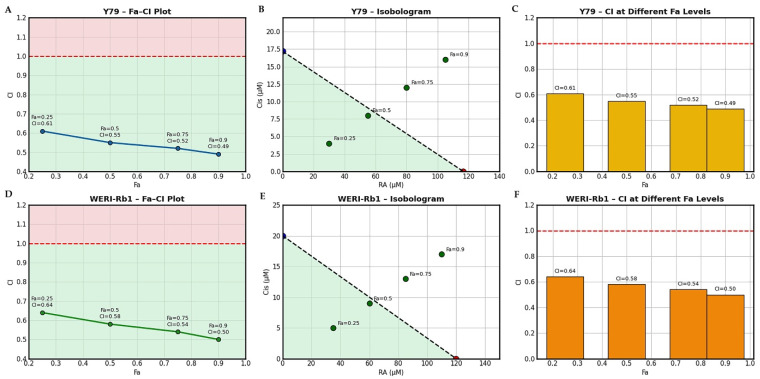
The synergistic interaction between RA and Cis was evaluated in Y79 and WERI-Rb1 RB cells using the Chou–Talalay method. (**A**–**C**) Combination analysis in Y79 cells. (**A**) Fa–CI plot illustrating the relationship between the fraction affected (Fa) and the corresponding CI values for the RA + Cis combination. CI values < 1 indicate synergism, CI = 1 indicates an additive effect, and CI > 1 indicates antagonism. (**B**) Isobologram analysis showing the IC_50_ values of RA and Cis as single agents and their combined doses at different Fa levels. The dashed line represents the line of additivity, and combination points located below this line indicate synergistic interactions. (**C**) Bar graph summarizing CI values at increasing Fa levels (Fa = 0.25, 0.5, 0.75, and 0.9), demonstrating dose-dependent synergism in Y79 cells. (**D**–**F**) Combination analysis in WERI-Rb1 cells. (**D**) Fa–CI plot showing consistent CI values below 1 across all evaluated Fa levels, indicating sustained synergistic interactions between RA and Cis. (**E**) Isobologram analysis depicting combination data points positioned below the line of additivity, further confirming synergy in WERI-Rb1 cells. (**F**) CI bar graph illustrating CI values at corresponding Fa levels, demonstrating robust synergism of the RA + Cis combination in WERI-Rb1 cells. The green shaded region represents synergism (CI < 1), whereas the red shaded region indicates antagonism (CI > 1). The dashed red line denotes the additivity threshold (CI = 1).

The interaction between RA and Cis in Y79 and WERI-Rb1 RB cells was quantitatively evaluated using the Chou–Talalay CI method. As shown in the Fa–CI plots (Figure 3A,D), CI values remained consistently below 1 across all examined fraction affected (Fa) levels (Fa = 0.25–0.9) in both cell lines, indicating a synergistic interaction between RA and Cis. In Y79 cells, CI values progressively decreased from 0.61 at Fa = 0.25 to 0.49 at Fa = 0.9, demonstrating increasing synergy with higher levels of growth inhibition. A similar trend was observed in WERI-Rb1 cells, where CI values declined from 0.64 to 0.50 over the same Fa range.

To ensure transparency of the Fa–CI analysis, the specific RA and Cis concentration pairs corresponding to the evaluated Fa levels were derived from fixed-ratio combination experiments based on their respective 48 h IC_50_ values. Increasing fractions of the IC_50_ (0.25×, 0.5×, 0.75×, 1×, and 2×) were applied to generate graded effect levels. The concentration pairs corresponding to Fa values of 0.25, 0.5, 0.75, and 0.9 were subsequently used for CI and DRI calculations according to the Chou–Talalay method.

Isobologram analysis further confirmed the synergistic interaction between RA and Cis in both cell lines (Figure 3B,E). The combination data points corresponding to different Fa levels were consistently positioned below the line of additivity, indicating that lower doses of each agent were required to achieve a given effect when used in combination compared to single-agent treatments. This effect was evident across low, intermediate, and high Fa levels, supporting robust synergy over a broad range of cytotoxic effects. The bar graphs summarizing CI values at distinct Fa levels (Figure 3C,F) illustrate that synergism was maintained throughout increasing effect levels in both Y79 and WERI-Rb1 cells. Notably, WERI-Rb1 cells exhibited slightly higher CI values at lower Fa levels compared to Y79 cells; however, both cell lines converged toward strong synergism at higher Fa values (Fa ≥ 0.75). Collectively, these findings demonstrate that the enhanced cytotoxicity observed with RA and Cis co-treatment is consistent with pharmacological synergy rather than simple additivity, and that this synergistic interaction is conserved across two distinct RB cell models (Figure 3).

To further characterize the interaction between RA and Cis, Bliss independence analysis was applied to MTT-derived viability data from Y79 and WERI-Rb1 RB cells. Heatmap visualization of Bliss excess scores revealed a clear concentration-dependent synergistic interaction between RA and Cis in both cell lines. In Y79 cells, synergism was primarily observed at higher RA concentrations (≥100 µM) combined with moderate to high Cis doses, whereas lower concentration combinations tended to display additive effects.

**Figure 4:**
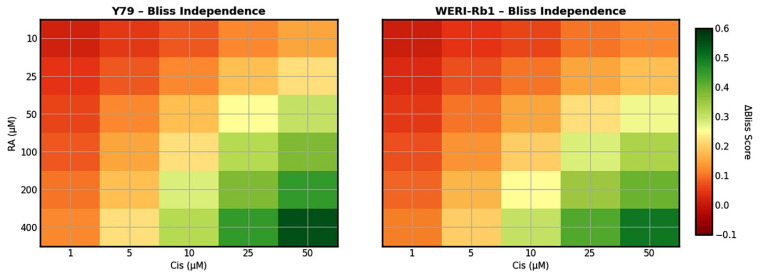
Bliss independence analysis of RA and Cis combination in Y79 and WERI-Rb1 RB cells. Bliss independence analysis was performed to evaluate the interaction between RA and Cis in Y79 and WERI-Rb1 RB cells using cell viability data obtained from the MTT assay. Heatmaps depict the Bliss excess scores (ΔBliss) calculated across a matrix of RA (10–400 µM) and Cis (1–100 µM) concentrations.

In contrast, WERI-Rb1 cells exhibited a comparable concentration-dependent synergy profile, with positive Bliss scores observed across multiple RA and Cis concentration pairs. This suggests a similar responsiveness of WERI-Rb1 cells to the combined treatment. Importantly, no substantial antagonistic interactions were detected in either cell line. Collectively, these findings corroborate CI analysis and demonstrate that the enhanced cytotoxicity observed with RA and Cis co-treatment is consistent with pharmacological synergism rather than simple additivity (Figure 4).

Quantitative assessment of Bliss excess scores further supported these findings. In Y79 cells, the mean ΔBliss value across the concentration matrix was approximately 0.23, with a maximum ΔBliss of 0.59 observed at RA 400 µM combined with Cis 50 µM. In WERI-Rb1 cells, a comparable mean ΔBliss value of 0.25 was detected, and the strongest synergistic interaction (ΔBliss = 0.57) was similarly observed at RA 400 µM and Cis 50 µM. Positive ΔBliss values increased progressively with higher RA and Cis concentrations, confirming a dose-dependent synergistic interaction consistent with CI analysis.

In addition, DRI analysis derived from the Chou–Talalay model indicated a favorable dose-sparing effect of the combination treatment. At higher Fa levels (Fa ≥ 0.75), the DRI values for Cis ranged from approximately 2.1 to 2.8 in Y79 cells and from 1.9 to 2.6 in WERI-Rb1 cells, indicating that equivalent levels of growth inhibition could be achieved using substantially lower Cis doses when combined with RA. Similarly, RA exhibited DRI values ranging from 1.5 to 2.0 across higher Fa levels in both cell lines. These findings suggest that the synergistic interaction between RA and Cis may allow dose reduction, particularly for Cis, while maintaining enhanced anticancer efficacy.

### 3.4. Quantitative Analysis of Apoptosis in Y79 and WERI-Rb1 Cells by Annexin V-FITC/PI Staining

The effects of RA, Cis, and their combination on apoptosis in Y79 RB and WERI-Rb1 cells were evaluated using Annexin V-FITC/PI double staining following 48 h of treatment. Flow cytometric analysis allowed the discrimination of four distinct cell populations: viable cells (Annexin V^−^/PI^−^), early apoptotic cells (Annexin V^+^/PI^−^), late apoptotic or secondary necrotic cells (Annexin V^+^/PI^+^), and necrotic cells (Annexin V^−^/PI^+^).

**Figure 5:**
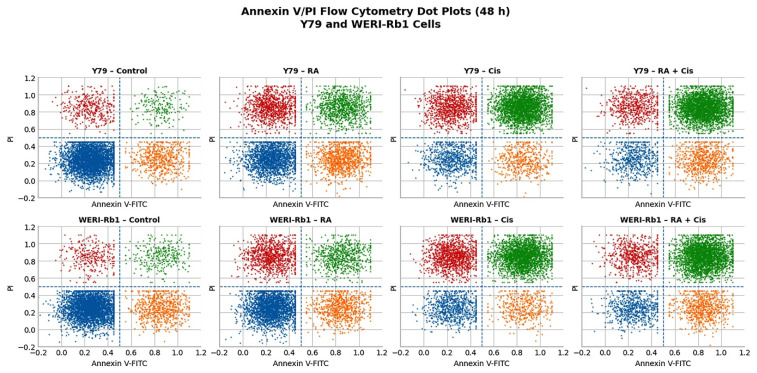
Representative Annexin V-FITC/PI flow cytometry dot plots showing apoptotic cell distribution in Y79 and WERI-Rb1 cells after 48 h treatment with RA, Cis, and their combination. The lower-left quadrant represents viable cells (Annexin V^−^/PI^−^), lower-right quadrant shows early apoptotic cells (Annexin V^+^/PI^−^), upper-right quadrant shows late apoptotic or necrotic cells (Annexin V^+^/PI^+^), and upper-left quadrant represents necrotic cells (Annexin V^−^/PI^+^). The combination treatment markedly increased both early and late apoptotic populations compared to single treatments (*p* < 0.001).

For Y79 cells, while the majority of cells in the control group were in the live quadrant (76%), the proportions of early apoptotic (12%), late apoptotic (4%), and necrotic (8%) cells were low. RA treatment led to a significant decrease in the live cell ratio (40.5%), while causing a significant increase in both early (19.5%) and late apoptotic (20%) cell populations. Cis treatment showed a strong cytotoxic effect on Y79 cells; the live cell ratio decreased to 15.5%, while late apoptotic cells became the dominant population (55.5%). The combined application of RA and Cis further increased the rate of late apoptotic cells to 62%, while reducing the viable cell population to 12%. These findings demonstrate that combined therapy more effectively induces apoptotic cell death in Y79 cells compared to single applications. For WERI-Rb1 cells, the viable cell rate was found to be 72% in the control group; early apoptotic (14%), late apoptotic (6%), and necrotic (6%) cells remained at limited levels. RA application reduced the viable cell rate to 50.5% and led to a significant increase, particularly in the rate of necrotic cells (20%). Cis treatment also showed a significant apoptotic effect in WERI-Rb1 cells; late apoptotic cells increased to 50.5%, while the rate of necrotic cells was found to be 24.5%. The combined administration of RA and Cis produced the highest apoptotic response, increasing the late apoptotic cell population to 59%, while the percentage of viable cells decreased to 14% (Figure 5). The presence of a small proportion of apoptotic/necrotic cells in control groups likely reflects baseline spontaneous cell death in suspension cultures.

### 3.5. Modulation of Tumor Cell-Derived Cytokine Release Following Single and Combination Treatments

**Figure 6:**
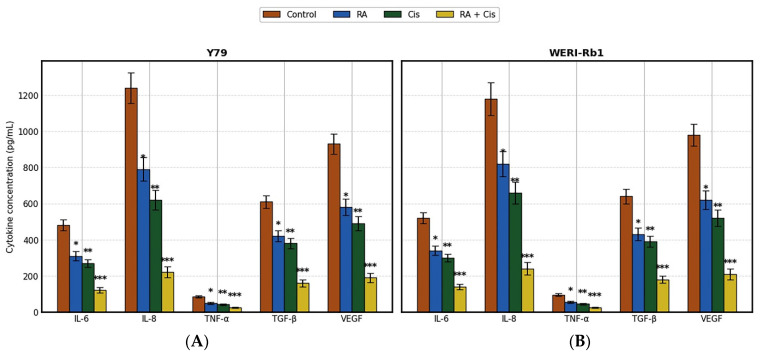
Cytokine secretion levels of IL-6, IL-8, TNF-α, TGF-β, and VEGF were quantified by ELISA in (**A**) Y79 and (**B**) WERI-Rb1 RB cells following 48 h treatment with RA, Cis, or their combination (RA + Cis). Data are expressed as mean ± SD of three independent experiments performed in triplicate. Statistical significance was determined by one-way ANOVA followed by Tukey’s post hoc test. Comparisons were made between control and treated groups. * *p* < 0.05, ** *p* < 0.01, *** *p* < 0.001 vs. control.

The effects of RA, Cis, and their combination on cytokine secretion were evaluated in Y79 and WERI-Rb1 RB cells after 48 h of treatment using ELISA (Figure 6). In both cell lines, treatment with RA or Cis alone significantly reduced the secretion of pro-inflammatory (IL-6, IL-8, TNF-α) and pro-angiogenic (VEGF) cytokines compared to control cells, while the combination treatment produced the most pronounced inhibitory effects.

In Y79 cells, RA treatment resulted in a moderate but significant decrease in IL-6 and IL-8 levels, whereas Cis exerted a stronger suppressive effect on these cytokines. Notably, the combined RA + Cis treatment led to a marked reduction in all measured cytokines, with IL-8 and VEGF levels decreasing to less than 25% of control values (*p* < 0.001). TNF-α secretion was also significantly diminished in the combination group, indicating enhanced anti-inflammatory activity. Similarly, WERI-Rb1 cells exhibited a significant reduction in cytokine secretion following RA or Cis treatment, with a response pattern comparable to that observed in Y79 cells. However, basal cytokine levels in WERI-Rb1 cells were generally higher than those in Y79 cells, particularly for IL-6, IL-8, and VEGF. Despite this, the RA + Cis combination induced a marked suppression of cytokine release, resulting in reductions of approximately 70–80% relative to control levels for IL-8 and VEGF (*p* < 0.001). Comparative analysis between the two cell lines revealed that WERI-Rb1 cells were slightly more resistant to single-agent RA or Cis treatment, whereas the combination therapy effectively overcame this difference, producing comparable levels of cytokine inhibition in both models. TGF-β secretion followed a similar trend, with moderate decreases after single treatments and a pronounced reduction following combination therapy in both Y79 and WERI-Rb1 cells. Overall, these findings indicate that the RA + Cis combination produces a more pronounced suppression of cytokine secretion than either agent alone in both Y79 and WERI-Rb1 cell lines, highlighting the potential of this strategy to modulate inflammatory and angiogenic signaling in RB (Figure 6).

### 3.6. Alterations in Intracellular ROS Levels Following Single and Combination Treatments

**Figure 7:**
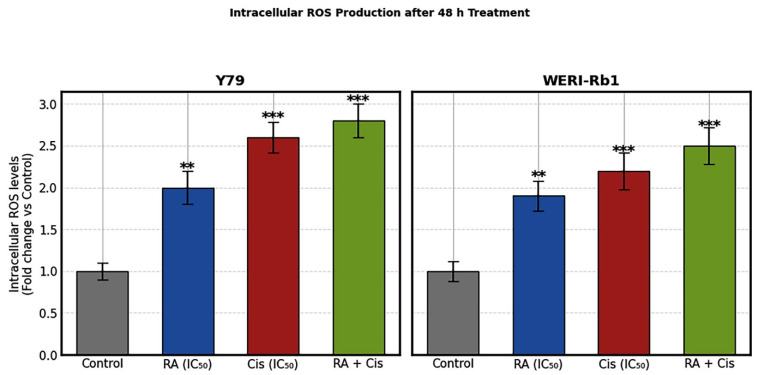
Intracellular ROS generation in Y79 and WERI-Rb1 cells after 48 h treatment. ROS levels were measured using the DCFH-DA assay and expressed as fold change relative to the control group. Data represent mean ± SD of three independent experiments (*n* = 3). Statistical significance was determined by one-way ANOVA followed by Tukey’s post hoc test: ** *p* < 0.01, *** *p* < 0.001 compared to control.

Intracellular ROS levels were quantified as a plate-based bulk measurement using DCF fluorescence in Y79 and WERI-Rb1 RB cells following treatment with RA, Cis, or their combination for 48 h. As shown in Figure 7, both agents significantly increased intracellular ROS production compared with untreated control cells in a cell line-dependent manner.

In Y79 cells, treatment with RA at its IC_50_ concentration resulted in an approximately 2.0 fold increase in intracellular ROS levels relative to the control group (*p* < 0.01). Cis treatment induced a more pronounced oxidative response, with ROS levels increasing approximately 2.6 fold compared to control cells (*p* < 0.001). Notably, the combination of RA and Cis produced the highest ROS generation, leading to an approximately 2.8 fold elevation in fluorescence intensity, which was significantly greater than that observed with either single-agent treatment (*p* < 0.001). A similar trend was observed in WERI-Rb1 cells, although the magnitude of ROS induction was slightly lower than that seen in Y79 cells. RA treatment increased ROS levels by approximately 1.9 fold (*p* < 0.01), while Cis treatment resulted in an approximately 2.2 fold increase relative to control cells (*p* < 0.001). Consistent with the Y79 findings, combined RA and Cis treatment elicited the strongest oxidative response in WERI-Rb1 cells, with ROS levels rising approximately 2.5 fold compared to control (*p* < 0.001). Comparative analysis revealed that Y79 cells were more sensitive to ROS induction by both single and combination treatments than WERI-Rb1 cells. Nevertheless, in both cell lines, the RA + Cis combination consistently induced significantly higher ROS levels than either RA or Cis alone. These findings suggest that enhanced oxidative stress contributes to the increased cytotoxic and pro-apoptotic effects observed with combined RA and Cis treatment in RB cells (Figure 7).

### 3.7. Caspase 3/7 Activity

**Figure 8:**
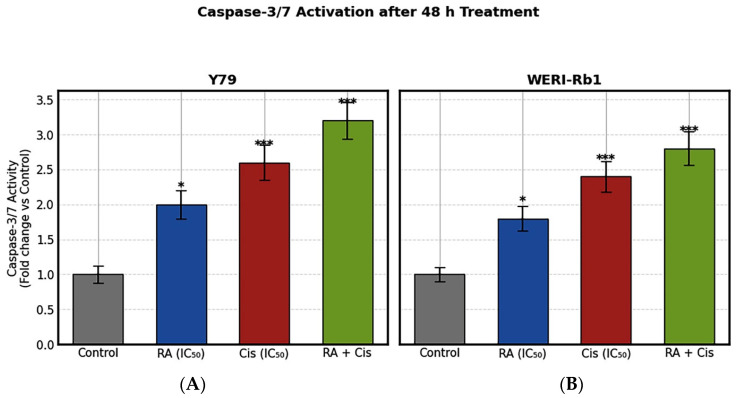
Activation of caspase-3/7 in Y79 and WERI-Rb1 RB cells following RA and Cis treatment. Caspase-3/7 activity was measured in (**A**) Y79 and (**B**) WERI-Rb1 RB cells after 48 h exposure to RA, Cis, or their combination (RA + Cis) at their respective IC_50_ concentrations. Caspase activity was determined using a luminescent assay and expressed as fold change relative to untreated control cells. Data are presented as mean ± SD of three independent experiments performed in triplicate. Statistical significance was assessed by one-way ANOVA followed by Tukey’s post hoc test. Comparisons were made between control and treated groups. * *p* < 0.05, *** *p* < 0.001 vs. control.

In Y79 cells, RA treatment at its IC_50_ concentration induced a significant increase in caspase-3/7 activity (approximately 1.9 fold vs. control, *p* < 0.01), indicating activation of early apoptotic signaling. Cis treatment resulted in a more pronounced activation, with caspase-3/7 levels increasing approximately 2.6 fold relative to the control group (*p* < 0.001). Notably, combined RA + Cis treatment produced the strongest apoptotic response, leading to an approximately 3.0 fold increase in caspase-3/7 activity, which was significantly higher than either single-agent treatment (*p* < 0.001). A similar trend was observed in WERI-Rb1 cells, although the magnitude of caspase activation was slightly lower compared to Y79 cells. RA increased caspase-3/7 activity by approximately 1.6 fold (*p* < 0.05), while Cis treatment elevated activity to approximately 2.2 fold over control levels (*p* < 0.001). The combination treatment again resulted in the highest caspase-3/7 activation, with an approximate 2.5 fold increase compared to control cells (*p* < 0.001) (Figure 8).

### 3.8. Modulation of Apoptosis-Related Gene Expression in Y79 Cells Following Single and Combination Treatments

**Figure 9:**
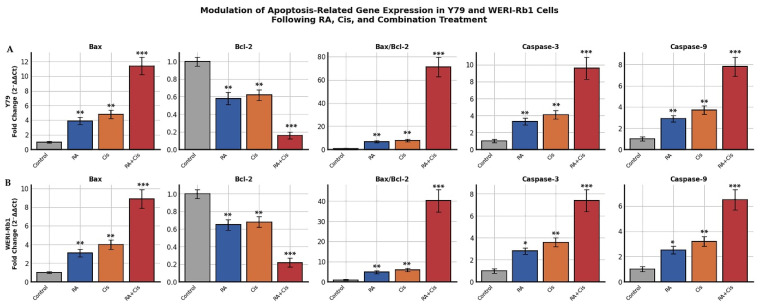
Analysis of apoptotic gene expression profile by qRT-PCR in Y79 cells. The mRNA expression levels of Bax, Bcl-2, Bax/Bcl-2 ratio, Caspase-3, and Caspase-9 were quantified in Y79 (**A**) and WERI-Rb1 (**B**) RB cells by qRT-PCR following treatment with RA, Cis, or their combination (RA + Cis) at their respective IC_50_ concentrations for 48 h. Gene expression levels were normalized to the housekeeping gene and calculated using the 2^−ΔΔCt^ method, with control values set to 1.0. Data are presented as mean ± SD of three independent experiments performed in triplicate. Combination treatment induced a pronounced upregulation of pro-apoptotic genes (Bax, Caspase-3, Caspase-9) and a marked downregulation of the anti-apoptotic gene Bcl-2 in both cell lines, resulting in a substantial increase in the Bax/Bcl-2 ratio, consistent with engagement of intrinsic apoptotic signaling. Y79 cells exhibited a stronger transcriptional response compared to WERI-Rb1 cells. Statistical significance was determined by one-way ANOVA followed by Tukey’s post hoc test. * *p* < 0.05, ** *p* < 0.01, *** *p* < 0.001 versus control.

In Y79 cells, treatment with either RA or Cis significantly upregulated the expression of the pro-apoptotic gene Bax, while the combined RA + Cis treatment induced a markedly stronger response. Bax mRNA levels increased approximately 3.9 fold and 4.8 fold following RA and Cis alone treatments, respectively, whereas combination treatment resulted in an approximately 11.4 fold increase compared to control cells (*p* < 0.001). In parallel, expression of the anti-apoptotic gene Bcl-2 was significantly downregulated, with the greatest suppression observed in the combination group (approximately 84% reduction vs. control, *p* < 0.001). Consistent with these findings, the Bax/Bcl-2 ratio, a critical indicator of mitochondrial apoptotic commitment, was significantly elevated in all treated groups, reaching a markedly elevated in the RA + Cis group (*p* < 0.001). This shift is consistent with engagement of intrinsic apoptotic signaling. Similarly, Caspase-3 and Caspase-9 mRNA expression levels were significantly increased following treatment, with combination therapy inducing the highest upregulation. Caspase-3 and Caspase-9 expression increased approximately 9.6 fold and 7.8 fold, respectively, in Y79 cells treated with RA + Cis, significantly exceeding the effects of single-agent treatments (*p* < 0.001). In WERI-Rb1 cells, a comparable expression pattern was observed. Although the magnitude of gene modulation was moderately lower than that seen in Y79 cells, combination treatment consistently resulted in significantly higher Bax, Bax/Bcl-2 ratio, Caspase-3, and Caspase-9 expression, along with pronounced suppression of Bcl-2. These findings are consistent with engagement of intrinsic (mitochondria-associated) apoptotic signaling in both RB cell lines, with Y79 cells displaying greater sensitivity to combination treatment (Figure 9).

### 3.9. Bioinformatics Analysis

#### 3.9.1. Protein–Protein Interaction Network Construction and KEGG Pathway Enrichment Analysis of Selected Target Proteins

To explore the interaction landscape of proteins associated with the experimental findings, a PPI network was constructed using the STRING database. The network included apoptosis- and inflammation-related proteins (Bax, Bcl-2, Caspase-3, IL-6, IL-8, TNF-α, TGF-β, and VEGF) and was generated using a high-confidence interaction score threshold (≥0.7).

**Figure 10:**
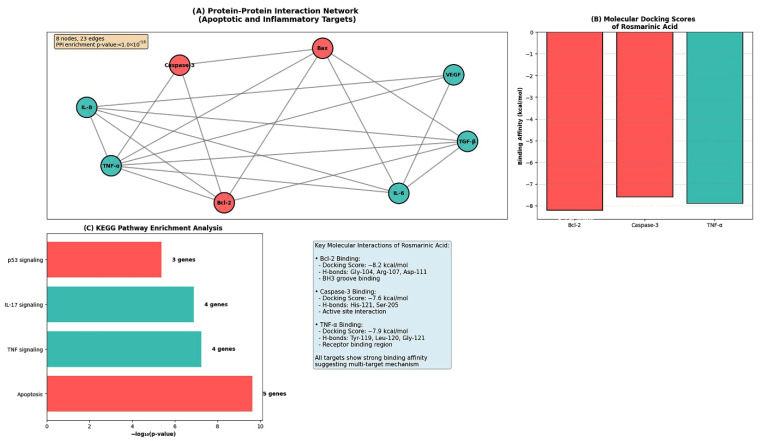
Bioinformatics analysis of target proteins. (**A**) Protein–protein interaction network of key apoptotic and inflammatory targets constructed using the STRING database. The network demonstrates significant connectivity among the selected proteins involved in apoptosis and inflammatory signaling. (**B**) Molecular docking scores of RA with selected target proteins, including Bcl-2, Caspase-3, and TNF-α, indicating favorable binding affinities. (**C**) KEGG pathway enrichment analysis of the selected targets, highlighting apoptosis, TNF signaling, IL-17 signaling, and p53 signaling pathways as significantly enriched biological processes associated with the identified protein network.

The resulting PPI network consisted of 8 nodes and 23 edges, with an average node degree of 5.75. The observed number of interactions was significantly higher than expected for a random set of proteins of similar size, as indicated by a PPI enrichment *p*-value of <1.0 × 10^−16^ (Table 2). The network topology is illustrated in Figure 10A.

KEGG pathway enrichment analysis was subsequently performed using the DAVID bioinformatics resource. Several pathways were found to be significantly enriched (false discovery rate, FDR < 0.05). Among these, the apoptosis pathway showed the highest level of enrichment, with 5 out of the 8 analyzed proteins associated with this pathway (Table 2).

#### 3.9.2. Molecular Docking Results of RA with Apoptosis- and Inflammation-Related Target Proteins

Molecular docking analyses were performed to evaluate the interaction of RA with selected apoptosis- and inflammation-related proteins, including Bcl-2, caspase-3, and TNF-α. Docking poses, binding affinities, and interaction profiles are summarized in Table 3, and representative docking conformations are shown in Figure 10A–C.

Docking Analysis with Bcl-2: RA docked within the Bcl-2 protein structure, forming hydrogen bond interactions with residues Gly-104, Arg-107, and Asp-111. In addition, π–π stacking interactions were observed with Phe-112, along with hydrophobic interactions involving Val-108, Ala-105, and Phe-112. The predicted docking pose of RA within the Bcl-2 structure is illustrated in Figure 10A.

Docking Analysis with Caspase-3: Docking of RA to caspase-3 yielded a predicted binding affinity of −7.6 kcal/mol. Hydrogen bond interactions were identified with residues His-121 and Ser-205. Additional interactions were observed between the catechol moiety of RA and the S1 subregion, while the carboxylic acid group interacted with residue Arg-207. The corresponding docking conformation is shown in Figure 10B.

Docking Analysis with TNF-α: RA exhibited a predicted binding affinity of −7.9 kcal/mol when docked to TNF-α. Hydrogen bond interactions were observed with residues Tyr-119, Leu-120, and Gly-121. Hydrophobic interactions involving Leu-57 and Tyr-59 were also identified. The docking pose and interaction pattern for the TNF-α– RA complex are presented in Figure 10C.

### 3.10. Evaluation of the Effects of RA and Cis in a 3D Tumor Spheroid Model

#### 3.10.1. Formation and Morphological Alterations of 3D Tumor Spheroids

**Figure 11:**
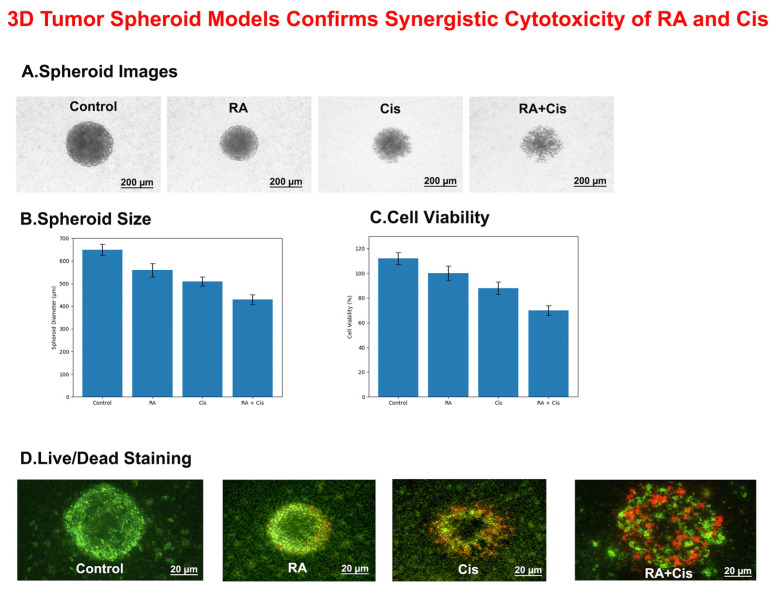
Evaluation of the synergistic effects of RA and Cis in a 3D tumor spheroid model of Y79 retinoblastoma cells. (**A**) Representative bright-field images of 3D tumor spheroids formed in ultra-low attachment plates following treatment with RA, Cis, or their combination (RA + Cis). Morphological alterations, reduction in spheroid diameter, and disruption of structural compactness were observed, particularly in the combination group. Scale bar: 200 µM. (**B**) Quantitative analysis of spheroid size expressed as spheroid diameter (µM). RA and Cis treatments reduced spheroid growth compared to control, while RA + Cis co-treatment resulted in the most pronounced inhibition of spheroid expansion. (**C**) Cell viability of 3D tumor spheroids assessed using an ATP-based CellTiter-Glo^®^ 3D assay. Viability is expressed as percentage of control. The combination treatment significantly decreased metabolic activity compared with single treatments. (**D**) Live/dead fluorescence staining of representative spheroids from control, RA, Cis, and RA + Cis groups. Live cells were stained with Calcein-AM (green), and dead cells were stained with Ethidium homodimer-1 (red). Increased red fluorescence and loss of spheroid integrity were most prominent in the RA + Cis group. Scale bar: 20 µM. Data are presented as mean ± SD (*n* = 3). Statistical significance was determined using one-way ANOVA followed by Tukey’s post hoc test.

To assess the effects of RA and Cis on tumor architecture, Y79 retinoblastoma cells were cultured as three-dimensional (3D) tumor spheroids using ultra-low attachment plates. Consistent with reduced drug penetration in 3D cultures, slightly higher concentrations were applied as described in the Methods. Under control conditions, cells formed compact, spherical, and well-defined spheroids with smooth borders. Treatment with RA resulted in a moderate reduction in spheroid size while largely preserving spheroid integrity. In contrast, Cis treatment induced a more pronounced decrease in spheroid size accompanied by partial disruption of spheroid compactness. Notably, combined RA and Cis treatment led to marked spheroid shrinkage and loss of structural integrity, characterized by irregular borders and fragmented morphology (Figure 11A).

#### 3.10.2. Quantitative Reduction of Spheroid Size Following RA and Cis Co-Treatment

Quantitative analysis of spheroid diameter demonstrated significant differences among treatment groups. Compared with control spheroids, RA treatment significantly reduced spheroid diameter, whereas Cis-induced a greater inhibitory effect. The most pronounced reduction in spheroid size was observed in the RA + Cis group, in which spheroid diameter was significantly lower than that observed in both single-agent treatment groups (*p* < 0.001). These findings are consistent with a potentiated growth-inhibitory effect under RA + Cis co-treatment in the 3D tumor spheroid model (Figure 11B).

#### 3.10.3. Suppression of Cell Viability in 3D Tumor Spheroids by RA and Cis Combination

Cell viability within 3D tumor spheroids was evaluated using an ATP-based CellTiter-Glo^®^ 3D assay. RA treatment alone resulted in a modest but significant reduction in spheroid viability compared with control. Cis treatment induced a more substantial decrease in cell viability. Importantly, combined RA and Cis treatment produced the strongest cytotoxic effect, significantly reducing cell viability relative to both control and single-agent treatments (*p* < 0.001). These results are consistent with the enhanced cytotoxic interaction observed in 2D cultures and demonstrate that the combined treatment remains effective under 3D conditions (Figure 11C).

#### 3.10.4. Live/Dead Fluorescence Staining Reveals Enhanced Cell Death in RA + Cis–Treated Spheroids

To further visualize treatment-induced cytotoxicity, live/dead fluorescence staining was performed on 3D tumor spheroids. Control spheroids predominantly exhibited green fluorescence, indicating a high proportion of viable cells. In contrast, spheroids treated with the RA and Cis combination displayed extensive red fluorescence, reflecting a substantial increase in non-viable cells. This was accompanied by a reduction in green fluorescence and partial disruption of spheroid architecture, supporting the enhanced cytotoxic effect of the combination treatment in a 3D tumor context (Figure 11D).

### 3.11. NAC Pretreatment Attenuates RA- and Cis-Induced ROS Generation

**Figure 12:**
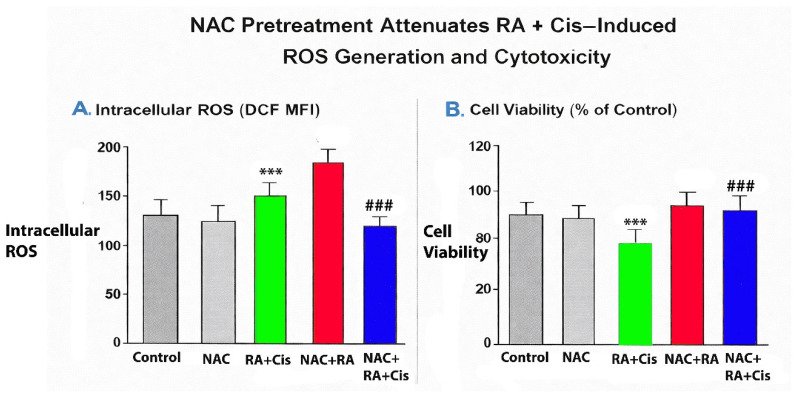
NAC pretreatment attenuates RA + Cis–induced ROS generation and cytotoxicity. Effects of NAC pretreatment on intracellular ROS levels and cell viability in retinoblastoma Y79 cells treated with RA and Cis. (**A**) Intracellular ROS levels quantified by DCF fluorescence and expressed as MFI. Treatment with RA + Cis significantly increased ROS levels compared with control, whereas NAC pretreatment markedly reduced ROS accumulation in RA + Cis–treated cells. NAC alone or in combination with RA did not significantly alter ROS levels relative to control. (**B**) Cell viability expressed as percentage of control following the indicated treatments. RA + Cis treatment significantly reduced cell viability, while NAC pretreatment partially reversed RA + Cis–induced cytotoxicity without fully restoring viability to control levels. Data are presented as mean ± SD from three independent experiments. Statistical significance was determined using one-way ANOVA followed by Tukey’s post hoc test. *** *p* < 0.001 vs. control; ^###^ *p* < 0.001 vs. RA + Cis.

Treatment with RA and Cis, particularly in combination, resulted in a significant increase in intracellular ROS levels compared with control cells. Notably, pretreatment with NAC markedly reduced ROS accumulation in RA + Cis–treated cells, as evidenced by a significant decrease in DCF fluorescence intensity (Figure 12A). NAC alone did not significantly alter intracellular ROS levels compared with control cells. Interestingly, the NAC + RA group demonstrated a modest rightward shift in DCF fluorescence intensity compared with control cells. This observation may reflect the context-dependent redox-modulatory properties of RA, which has been reported to exert mild pro-oxidant activity under certain intracellular conditions. NAC pretreatment may influence intracellular thiol balance and redox cycling dynamics, potentially modulating RA-induced ROS responses. However, the magnitude of ROS increase in the NAC + RA group remained substantially lower than that observed in the RA + Cis combination, indicating that NAC effectively attenuates excessive oxidative stress induced by dual treatment.

To validate ROS modulation at the single-cell level and to determine whether changes in bulk fluorescence were reflected in population-wide shifts, flow cytometry–based ROS analysis was performed following NAC pretreatment. Representative histograms demonstrated a clear rightward shift in DCF fluorescence intensity after RA + Cis treatment, which was substantially attenuated by NAC pretreatment (Figure 12B).

**Figure 13:**
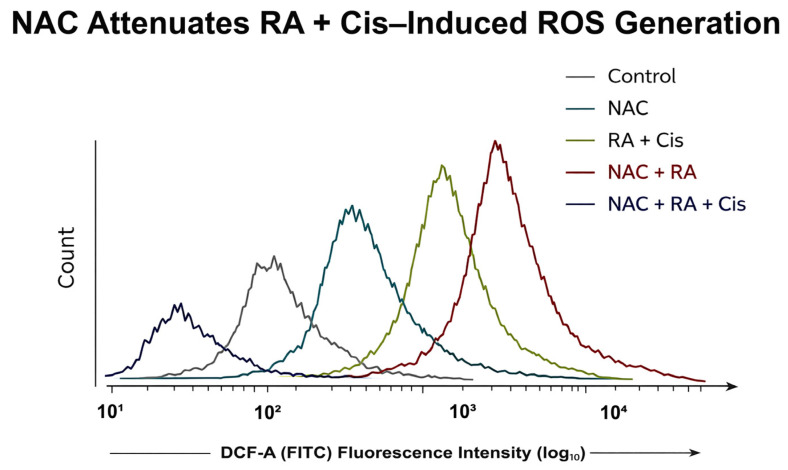
Representative flow cytometry histograms showing the effect of NAC pretreatment on RA + Cis–induced ROS generation in Y79 cells. Intracellular ROS levels were assessed using DCF fluorescence. RA + Cis treatment induced a rightward shift in fluorescence intensity, indicating increased ROS production, whereas NAC pretreatment attenuated this shift, demonstrating effective ROS scavenging. Histograms are representative of three independent experiments.

To further validate ROS modulation at the single-cell level, representative flow cytometry histograms of DCF fluorescence were analyzed following NAC pretreatment (Figure 13). Consistent with the quantitative MFI data, RA + Cis treatment induced a clear rightward shift in DCF fluorescence intensity compared with control cells, indicating increased intracellular ROS accumulation. NAC pretreatment attenuated this shift in RA + Cis–treated cells, demonstrating effective ROS scavenging. NAC alone did not produce a substantial change in fluorescence distribution relative to control. These findings confirm that the observed ROS modulation occurs at the single-cell level and is not solely attributable to population-averaged fluorescence measurements.

#### 3.11.1. ROS Scavenging Partially Reverses RA + Cis–Induced Cytotoxicity

Consistent with the observed reduction in ROS levels, NAC pretreatment significantly attenuated the cytotoxic effects of RA and Cis combination treatment. Cells pretreated with NAC exhibited higher viability compared with cells treated with RA + Cis alone, although viability did not fully return to control levels. These findings indicate that ROS generation contributes, at least in part, to the cytotoxic effects induced by RA and Cis co-treatment (Figure 12B).

#### 3.11.2. ROS Contributes to, but Does Not Fully Account for, RA + Cis–Induced Cell Death

Although NAC pretreatment significantly reduced both ROS accumulation and cell death, the incomplete rescue of cell viability suggests that additional ROS-independent mechanisms may also be involved in RA + Cis–induced cytotoxicity. This observation supports a multifactorial mode of action underlying the synergistic effects of RA and Cis.

## 4. Discussion

This study provides evidence that RA, a naturally occurring polyphenolic compound, synergistically enhances the anticancer efficacy of Cis in retinoblastoma cells through multiple complementary molecular mechanisms. Importantly, this synergistic interaction was consistently observed in both Y79 and WERI-Rb1 cell lines under two-dimensional culture conditions, supporting the robustness and broader relevance of the combination strategy beyond a single cellular model. Beyond conventional monolayer assays, the synergistic cytotoxic effects of RA and Cis were further validated in a three-dimensional tumor spheroid model, where combination treatment led to pronounced spheroid shrinkage, loss of structural integrity, and reduced cell viability. These findings indicate that the observed synergy is preserved in a more physiologically relevant tumor architecture. Mechanistically, the combination-induced cytotoxicity was closely associated with enhanced intracellular oxidative stress and activation of apoptotic signaling pathways. Functional rescue experiments using NAC demonstrated that scavenging of reactive oxygen species significantly attenuated both ROS accumulation and cell death, indicating that oxidative stress contributes substantially, but not exclusively, to the cytotoxic effects of RA and Cis co-treatment. Together, these results support a multifactorial mode of action in which oxidative stress–mediated apoptosis, in conjunction with additional ROS-independent mechanisms, underlies the synergistic anticancer activity of the RA and Cis combination.

The time- and dose-dependent cytotoxic effects observed in MTT assays established a strong rationale for combining RA with Cis in both cell lines. Cis exhibited potent cytotoxicity in Y79 and WERI-Rb1 cells, with IC_50_ values decreasing markedly over time, consistent with its well-established mechanism as a DNA-damaging platinum-based chemotherapeutic agent. RA displayed moderate but significant antiproliferative activity in both models; however, Y79 cells consistently exhibited greater sensitivity to RA compared to WERI-Rb1 cells, suggesting intrinsic differences in redox balance, apoptotic threshold, or metabolic capacity between the two RB subtypes. Despite these differences, CI and Fa–CI analyses revealed a consistent synergistic interaction across the evaluated Fa levels in both cell lines, indicating that RA enhances Cis-induced cytotoxicity irrespective of baseline sensitivity. These findings highlight the potential of RA to act as a chemosensitizer and raise the possibility of reducing Cis dosage in future therapeutic strategies, an approach that could mitigate Cis-associated toxicity while preserving antitumor efficacy [17]. Nevertheless, this possibility must be rigorously validated in appropriate in vivo toxicity and efficacy models prior to clinical translation.

Mechanistically, the enhanced cytotoxicity of the RA + Cis combination was associated with transcriptional and functional changes consistent with activation of the intrinsic apoptotic signaling pathway in both Y79 and WERI-Rb1 cells. Combination treatment induced a pronounced increase in the Bax/Bcl-2 ratio, a widely used molecular indicator of intrinsic apoptotic signaling and apoptotic commitment [18]. Although this effect was observed in both cell lines, the magnitude of Bax/Bcl-2 ratio elevation was markedly higher in Y79 cells, indicating a more pronounced shift toward a pro-apoptotic state. This difference likely reflects cell line specific variations in anti-apoptotic buffering capacity or mitochondrial priming. Notably, marked suppression of Bcl-2 expression may disproportionately elevate the Bax/Bcl-2 ratio, reflecting an apoptotic bias rather than absolute Bax overexpression [19]. These molecular alterations were supported by flow cytometric analyses, which demonstrated substantially higher proportions of both early and late apoptotic cells in the combination group compared to single-agent treatments. Although caspase-3 activation is ultimately a post-translational event, increased caspase-3 mRNA expression reflects transcriptional regulation associated with apoptotic signaling. In the present study, apoptosis was therefore evidenced at both the cellular level, through Annexin V/PI staining, and the molecular level, via modulation of Bax/Bcl-2 ratios and Caspase-3 and Caspase-9 mRNA expression. Collectively, these findings are consistent with engagement of intrinsic apoptotic signaling. Although caspase-3/7 activity was directly confirmed in this study, additional protein-level validation (e.g., Western blot analysis) could further strengthen mechanistic interpretation.

In silico bioinformatics and molecular docking analyses provided complementary structural and network-level insights that were broadly consistent with the experimental findings in both RB models. Protein–protein interaction and pathway enrichment analyses highlighted apoptosis-, TNF-, and p53-related signaling as central nodes targeted by the combination treatment. Docking simulations suggested that RA may interact with key regulators such as Bcl-2, caspase-3, and TNF-α, offering plausible molecular explanations for its chemosensitizing effects. While these computational predictions require experimental validation, they support a model in which RA lowers the apoptotic threshold and modulates inflammatory signaling, thereby sensitizing both Y79 and WERI-Rb1 cells to Cis-induced cytotoxicity [20].

An additional mechanistic layer contributing to the observed synergy involves oxidative stress. Combination treatment resulted in significantly elevated intracellular ROS levels in both Y79 and WERI-Rb1 cells, with Y79 cells again exhibiting a stronger response. ROS are well-known upstream regulators of mitochondrial dysfunction and apoptotic signaling, and their accumulation likely acts as a critical trigger linking RA-mediated redox modulation with Cis-induced DNA damage. The convergence of ROS induction, Bax/Bcl-2 imbalance, and caspase activation underscores a tightly coordinated apoptotic response elicited by the combination therapy.

Beyond apoptosis, the RA + Cis combination exerted a pronounced suppressive effect on pro-inflammatory and pro-angiogenic cytokines, including IL-6, IL-8, TNF-α, VEGF, and TGF-β, in both cell lines. Tumor-derived cytokines are increasingly recognized as key contributors to RB progression by promoting tumor cell survival, angiogenesis, and resistance to therapy. The consistent downregulation of these factors in Y79 and WERI-Rb1 cells suggests that RA + Cis not only induces direct tumor cell death but also disrupts autocrine and paracrine signaling networks that support tumor growth. Notably, cytokine suppression was more pronounced in Y79 cells, paralleling their heightened apoptotic and oxidative stress responses, and further emphasizing cell line-specific differences in treatment susceptibility [21,22]. The marked reduction of these factors at the tumor cell level suggests that the combination therapy may not only directly induce tumor cell death but also attenuate autocrine and paracrine signaling pathways that support tumor growth and dissemination. Further in vivo studies are necessary to determine the broader biological and therapeutic implications of this cytokine modulation.

The observed synergy between RA and Cis may arise from multiple, non-mutually exclusive mechanisms. RA may enhance Cis uptake or interfere with DNA repair processes, thereby amplifying Cis-induced DNA damage. Additionally, as suggested by the docking analyses, RA may directly target anti-apoptotic proteins such as Bcl-2, effectively lowering the apoptotic threshold and sensitizing cells to Cis-induced death signals. Concurrent suppression of pro-inflammatory and pro-angiogenic gene expression and cytokine release further creates an intracellular signaling milieu unfavorable for RB cell survival. In line with these observations, natural polyphenols such as RA have been reported to synergize with various chemotherapeutic agents, including platinum-based compounds, across multiple cancer models [23,24,25].

From a clinical perspective, these findings suggest that RA may represent a promising adjunct to Cis-based chemotherapy for RB. If the observed synergy is validated in vivo, achieving comparable or enhanced therapeutic efficacy at lower Cis doses could potentially reduce the nephrotoxicity, neurotoxicity, and ototoxicity associated with Cis monotherapy [26,27]. Moreover, the multi-target activity of RA, affecting both tumor cells and their associated inflammatory and angiogenic signaling networks, is consistent with contemporary cancer treatment paradigms that aim to simultaneously target multiple hallmarks of cancer [28].

**Figure 14:**
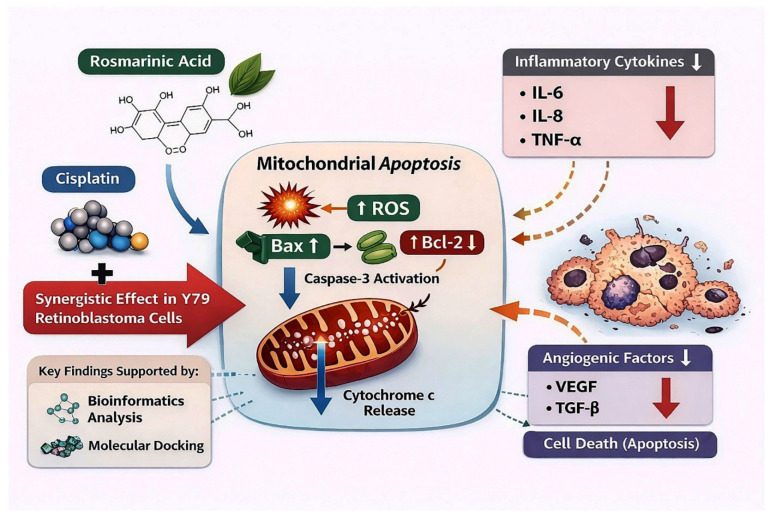
Schematic summary of the synergistic effects of RA and Cis in RB cells, highlighting the coordinated induction of oxidative stress, engagement of intrinsic apoptosis signaling, and suppression of tumor cell–derived inflammatory and angiogenic signals.

Taken together, the experimental and in silico findings of this study are supported by protein–protein interaction network analysis and molecular docking studies presented in Figure 10. As illustrated, the combination of RA and Cis exerts a coordinated, multi-level anticancer effect in Y79 and WERI-Rb1 cells. At the cellular level, combined treatment results in enhanced intracellular ROS generation, which is accompanied by a pronounced shift in the Bax/Bcl-2 balance toward a pro-apoptotic state, activation of initiator and effector caspases, and a marked increase in apoptotic cell populations. In parallel, the combination robustly suppresses the release of tumor cell-derived and pro-angiogenic cytokines, including IL-6, IL-8, TNF-α, VEGF, and TGF-β, thereby potentially attenuating autocrine signaling pathways that support tumor survival and progression. In silico protein–protein interaction and molecular docking analyses provide additional structural and network-level context, suggesting that RA may interact with key regulators of apoptotic and inflammatory signaling. Although these computational findings do not constitute functional proof, they support the experimentally observed convergence of oxidative stress induction, engagement of intrinsic (mitochondria-associated) apoptotic signaling, and cytokine suppression. Collectively, Figure 14 provides a schematic summary of the multifaceted and synergistic effects of the RA + Cis combination in Y79 and WERI-Rb1 cells.

Clinically, the demonstration of synergistic efficacy across two distinct RB cell lines strengthens the translational relevance of the RA + Cis combination. The ability of RA to enhance Cis activity while potentially allowing dose reduction is particularly attractive given the severe systemic toxicities associated with Cis-based chemotherapy. Moreover, the multi-target nature of RA simultaneously affecting oxidative stress, apoptosis, inflammation, and angiogenesis aligns well with contemporary therapeutic strategies aimed at targeting multiple hallmarks of cancer.

Despite these promising findings, several limitations should be acknowledged. Although the inclusion of both Y79 and WERI-Rb1 cells enhances the generalizability of the two-dimensional in vitro findings, retinoblastoma is a heterogeneous disease, and additional cell lines as well as patient-derived models should be investigated in future studies. Importantly, the present experiments were conducted in treatment-naïve RB cell lines and therefore do not directly model acquired or intrinsic chemoresistance, which represents a major clinical challenge in advanced retinoblastoma management. Future studies should evaluate the efficacy of RA in chemoresistant RB models and in combination with clinically established frontline regimens to better define its translational potential. Although caspase-3/7 enzymatic activity was directly quantified in the present study, protein-level validation of cleaved caspase-3 and cleaved caspase-9 would further strengthen mechanistic resolution. Moreover, although modulation of the Bax/Bcl-2 ratio and caspase-9 expression is consistent with involvement of the intrinsic apoptotic pathway, direct assessment of mitochondrial structure, mitochondrial DNA content, or mitochondrial biogenesis markers was not performed in the present study. Future investigations incorporating ultrastructural analyses and functional mitochondrial assays would provide a more comprehensive characterization of mitochondrial involvement. In addition, although the synergistic effects of the RA and Cis combination were confirmed in a three-dimensional tumor spheroid model, in vivo validation remains essential to assess pharmacokinetics, bioavailability, systemic toxicity, and therapeutic efficacy. Importantly, whether the observed in vitro synergy translates into reduced Cis-associated toxicity in vivo remains an unanswered question. Furthermore, the absence of non-tumoral retinal or ocular epithelial cell controls represents an additional limitation of the present study. Future investigations should evaluate the selective cytotoxicity and ROS modulation of the RA + Cis combination in normal retinal cell models to better define the therapeutic window and assess potential off-target toxicity.

In addition, the bioinformatics and molecular docking analyses presented in this study provide predictive structural and pathway-level insights but do not constitute functional proof of direct molecular interactions. Experimental validation of these in silico predictions, such as target-specific inhibition studies, co-immunoprecipitation assays, or binding affinity measurements, will be necessary. Future studies should therefore focus on in vivo efficacy and safety evaluations, functional validation of apoptotic signaling pathways at the protein level, and optimization of dosing strategies. Collectively, addressing these limitations will be critical for advancing this combination approach toward clinical relevance in retinoblastoma therapy.

## 5. Conclusions

In conclusion, the present study shows that RA enhances the anticancer activity of Cis in human retinoblastoma cells, with comparable effects observed in both Y79 and WERI-Rb1 models under two-dimensional culture conditions. Synergistic interactions were quantitatively supported using complementary analytical approaches, including the Chou–Talalay CI method, Bliss independence analysis, and DRI evaluation, demonstrating CI values below 1, positive ΔBliss scores, and favorable dose-reduction indices across multiple Fa levels. These findings support the potential role of RA as a chemosensitizing agent in this experimental setting.

Beyond monolayer assays, the combined effects of RA and Cis were preserved in a three-dimensional tumor spheroid model, where co-treatment was associated with spheroid shrinkage, reduced structural integrity, and decreased viability, supporting the biological relevance of this interaction in a more physiologically representative context. Mechanistically, combination treatment was associated with increased intracellular ROS levels, modulation of apoptosis-related signaling markers, and reduced tumor cell-derived cytokine secretion. NAC rescue experiments further suggest that oxidative stress contributes, at least in part, to the observed cytotoxic effects.

Collectively, these in vitro findings suggest that RA may represent a promising adjunct candidate for retinoblastoma therapy. DRI analysis indicates that combination treatment may permit effective growth inhibition at lower Cis concentrations in vitro. However, in vivo validation and further mechanistic investigations are required to confirm translational applicability and safety.

## Figures and Tables

**Table 1:** Primer sequences used in qRT-PCR.

Gene	Forwad Primer (5′→3′)	Reverse Primer (5′→3′)
*BAX*	TCAGGATGCGTCCACCAAGAAG	TGTGTCCACGGCGGCAATCATC
*BCL2*	ATCGCCCTGTGGATGACTGAGT	GCCAGGAGAAATCAAACAGAGGC
*CASP3*	GGAAGCGAATCAATGGACTCTGG	GCATCGACATCTGTACCAGACC
*CASP9*	GTTTGAGGACCTTCGACCAGCT	CAACGTACCAGGAGCCACTCTT
*ACTB*	CATTGCTGACAGGATGCAGAAGG	TGCTGGAAGGTGGACAGTGAGG

**Table 2:** Significantly enriched KEGG pathways for the target proteins.

Pathway	Count	%	*p*-Value	FDR
Apoptosis	5	62.5	2.4 × 10^−10^	3.2 × 10^−9^
TNF signaling pathway	4	50.0	5.7 × 10^−8^	3.8 × 10^−7^
IL-17 signaling pathway	4	50.0	1.3 × 10^−7^	5.8 × 10^−7^
p53 signaling pathway	3	37.5	4.2 × 10^−6^	1.4 × 10^−5^

**Table 3:** Molecular docking results of RA with target proteins.

Target Protein	PDB ID	Binding Affinity (kcal/mol)	Hydrogen Bonds
Bcl-2	1G5M	−8.2	3
Caspase-3	1GFW	−7.6	2
TNF-α	2AZ5	−7.9	3

## Data Availability

The original contributions presented in this study are included in the article. Further inquiries can be directed to the corresponding authors.

## Abbreviations

RB	Retinoblastoma
RA	Rosmarinic Acid
Cis	Cisplatin
Y79	Human retinoblastoma Y79 cell line
MTT	3-(4,5-Dimethylthiazol-2-yl)-2,5-diphenyltetrazolium bromide
CI	Combination Index
ROS	Reactive Oxygen Species
ELISA	Enzyme-Linked Immunosorbent Assay
qRT-PCR	Quantitative Real-Time Polymerase Chain Reaction
Bax	Bcl-2-associated X protein
Bcl-2	B-cell lymphoma 2
IL-6	Interleukin-6
IL-8	Interleukin-8
TNF-α	Tumor Necrosis Factor-alpha
TGF-β	Transforming Growth Factor-beta
VEGF	Vascular Endothelial Growth Factor
DMSO	Dimethyl Sulfoxide
PBS	Phosphate-Buffered Saline
FBS	Fetal Bovine Serum
PI	Propidium Iodide
DCFH-DA	2′,7′-Dichlorofluorescin Diacetate
IC_50_	Half-Maximal Inhibitory Concentration
Fa	Fractional Effect
PPI	Protein–Protein Interaction
KEGG	Kyoto Encyclopedia of Genes and Genomes
STRING	Search Tool for the Retrieval of Interacting Genes/Proteins
DAVID	Database for Annotation, Visualization, and Integrated Discovery
PDB	Protein Data Bank
BH3	Bcl-2 Homology 3
ANOVA	Analysis of Variance
SD	Standard Deviation
